# Synthesis and Statistical Optimization of Poly (Lactic-Co-Glycolic Acid) Nanoparticles Encapsulating GLP1 Analog Designed for Oral Delivery

**DOI:** 10.1007/s11095-019-2620-9

**Published:** 2019-05-13

**Authors:** Ruba Ismail, Tamás Sovány, Attila Gácsi, Rita Ambrus, Gábor Katona, Norbert Imre, Ildikó Csóka

**Affiliations:** 1 University of Szeged, Faculty of Pharmacy, Institute of Pharmaceutical Technology and Regulatory Affairs, Eötvös u. 6, Szeged, H-6720 Hungary; 2 University of Szeged, Faculty of Pharmacy, Institute of Pharmaceutical Analysis, Somogyi u. 4, Szeged, H-6720 Hungary

**Keywords:** liraglutide, oral delivery, plackett Burman design, PLGA nanoparticles, quality by design

## Abstract

**Purpose:**

To design and stabilize Liraglutide loaded poly (lactic-co-glycolic acid) nanoparticles (PLGA NPs) proper for oral administration.

**Methods:**

PLGA NPs were prepared by means of double emulsion solvent evaporation method and optimized by applying 7-factor 2-level Plackett-Burman screening design.

**Results:**

Spherical shaped NPs with homogeneous distribution, 188.95 nm particle size and 51.81% encapsulation efficiency were obtained. Liraglutide was successfully entrapped in the NPs while maintaining its native amorphous nature, and its structural integrity as well.

**Conclusion:**

Lira-PLGA NPs with the required Critical Quality Attributes (CQAs) were successfully designed by implementing a 7-factor 8-run Plackett Burman design into the extended Quality by Design (QbD) model, to elucidate the effect of formulation and process variables on the particle size, size-distribution, encapsulation efficiency and surface charge. As the developed nanoparticles maintained the native structure of the active pharmaceutical ingredient (API), they are promising compositions for the further development for the oral delivery of Lira.

**Figure:**
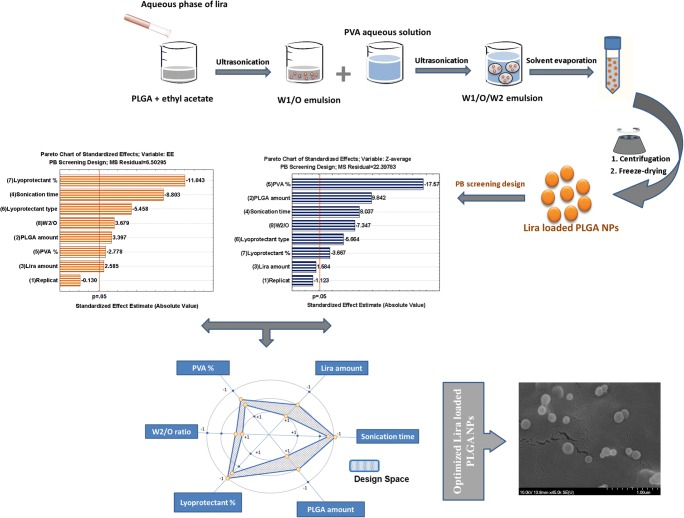
Graphical Abstract

**Electronic supplementary material:**

The online version of this article (10.1007/s11095-019-2620-9) contains supplementary material, which is available to authorized users.

## Introduction

A current scenario in pharmaceutical development is inclined towards employing rational Quality by Design (QbD) strategy (1,2) which has been adopted by the pharmaceutical industry to guarantee the quality of drug products (3). One of the key elements of QbD is to identify and thoroughly understand formulation and process variables and their effects on the critical quality attributes (CQAs), followed by the optimization of these variables by applying an appropriate statistical design of experiment (DOE) which enables the researcher to minimize the number of runs and helps in identifying the most influential parameters namely; critical process parameters (CPPs) and critical material attributes (CMAs) which may highly impact the quality of the product. In addition to that, DOE helps in identifying the optimum level of each factor that assures the desired CQAs values, to comply with the desired Quality Target Product Profile (QTPP) (4). One of the important questions when implementing DoE methodology is the selection of adequate experimental design.that matches the experimental objective. When estimating the main effects of large number of factors are of interest to be investigated, screening designs such as 2- level Plackett-Burman (PB) is applied. The main advantage of applying such screening designs is the minimum number of observations needed to calculate the effect of several variables. If providing further information on direct and pairwise-interaction effects and curvilinear variable effects is desired, second order designs: central composite designs (CCD) and Box Behncken designs (BBD) are the most widely applied ones (5,6). CCD provides better prediction capability than BBD, while the latter requires fewer runs in case of 3 or 4 variables and is applied when combined factor extremes should be avoided.

Liraglutide (Lira) or NN2211 is a recombinant palmityl-acylated derivative of glucagon like peptide −1 (GLP1), which was approved for the treatment of T2DM (6) in addition to chronic weight management (7). Lira is currently administered once daily through subcutaneous injection which is an invasive route known to be limited by insufficient patient adherence to the therapy in addition to the fact that this therapeutic route is not strictly mimicking the physiological secretion route of GLP1 (8). The oral route should be regarded as the most desirable choice for the delivery of Lira (9), and realizing the dream of administering a GLP1 analog such as Lira orally is still an elusive goal despite all advances in peptide delivery systems.

Based on our careful evaluation of literature regarding the emerging developments in oral delivery of antidiabetic peptides (10,11), we found out that PLGA nanoparticles showed promising results in improving the stability of peptides through the GIT in addition to other merits of nanocarrier systems, which can all lead to enhancing the oral bioavailability of these peptides (12). Among the applied techniques for preparing PLGA NPs, the double emulsion solvent evaporation method was found to be the most preferable one, which has been efficiently used for encapsulating peptides and proteins (13–15). Nevertheless, the physicochemical properties of nanoparticles may be affected by various formulation and process parameters, which influence the product quality.

To the best of our knowledge, there is no previously reported work considering the application of DOE as a part of the QbD strategy for the development of PLGA NPs encapsulating GLP-1 analog. Here we focus on the optimization of the size and EE of the NPs as a crucial demand along with the polydisperisity index (PDI) and surface charge. The design space (DS) was established to optimize the level of each of the examined factors, then surface morphology, compatibility studies as well as structural and conformational stability tests of Lira encapsulated in the optimized formula were conducted on the optimized formula.

## Materials and Methods

### Materials

Liraglutide was purchased from Xi’an Health Biochem Technology Co., Ltd. (China), Poly(lactide-co-glycolide) (PLGA 50:50, Mw = 30,000–60,000 Da), PVA (MOWIOL 4–98, MW~27,000 Da) which is a soluble polymer, and D-(+)-Trehalose dihydrate (MW = 378.33 g/mol) were purchased from Sigma Aldrich (Germany). D-(−)-Mannitol was purchased from Molar Chemicals Ltd. (Hungary). Sodium acetate anhydrous was purchased from Scharlau Chemie S.A. (Spain). Ethyl acetate used for dissoloving PLGA was from REANAL Labor (Hungary). All other chemicals in the study were of analytical reagent grade.

### Methods

#### Preparation of Liraglutide Loaded PLGA NPs Using Double Emulsion Solvent Evaporation Method

The preparation of Liraglutide loaded PLGA nanoparticles was carried out by means of the double emulsion W1/O/W2-solvent evaporation method, which is the most commonly used technique for the encapsulation of peptide drugs within PLGA NPs due to its simplicity and high encapsulation efficiency (15,16). The amount of PLGA (30 or 60 mg) was dissolved in ethyl acetate at room temperature to form the organic phase. Ethyl acetate was the organic solvent of choice here as it was reported to increase the rate of encapsulation of hydrophilic molecules (16). The inner aqueous phase of 0.5–5 mg liraglutide was dissolved in 0.5 ml of 1% sodium acetate aqueous solution, it was slowly added to the organic phase, then water/oil primary emulsion was formed upon sonication at the power of 90 W for 30 s using a probe sonicator in ice bath. The obtained emulsion was re-emulsified with external aqueous phase containing 0.5–2% PVA as stabilizer by sonication in ice bath at the power of 90 W for 0.5–2 min using the probe sonicator. The obtained water-in-oil-in-water (W1/O/W2) double emulsion was subjected to magnetic stirring at room temperature over the night to allow the complete evaporation of ethyl acetate. The nanoparticles were then collected by centrifugation for 15 min at 16500 rpm, washed three times with distilled water, and resuspended in deionized water.

For the lyophilization process, 1.5 ml of each nanoparticles suspension was poured into semi-stoppered glass vials with slotted rubber closures and freeze-dried at −40°C for 72 h. 5–10% mannitol or trehalose was added as lyoprotectants. The chamber pressure was maintained at 0.01 mbar, and the process was controlled by (Scanlaf CTS16a02) software.

#### Design of Experiment Study Using Plackett Burman Design

PB design is the most widely used method among the various screening designs used for the determination of the most influential factors affecting the pharmaceutical development as it has many advantages: it screens a large number of variables and identifies the highly influential ones with relatively few runs, while assuring a good degree of accuracy. PB design with a total of 8 runs involving 7 independent variables was carried out using STATISTICA 13 software, and analysis of variance (ANOVA) was applied to determine the statistical significance of each model coefficient, which was significant at 95% level (*P* < 0.05).

The linear equation of this model is:

$$ \mathrm{Y}={\mathrm{b}}_0+{\mathrm{b}}_1{\mathrm{X}}_1+{\mathrm{b}}_2{\mathrm{X}}_2+{\mathrm{b}}_3{\mathrm{X}}_3+{\mathrm{b}}_4{\mathrm{X}}_4+{\mathrm{b}}_5{\mathrm{X}}_5+{\mathrm{b}}_6{\mathrm{X}}_6+{\mathrm{b}}_7{\mathrm{X}}_7 $$

where Y is the response, b_0_ is the constant and b_1_, b_2_…b_7_ are the coefficient of factors X_1_, X_2_…X_7_. It is known that a positive value of the regression coefficient is an indicator of a positive effect of the factor (X) on the response (Y), while a negative value refers to an inverse relation between the examined variable and the response (17).

Depending on our previous risk assessment-based investigation (18–20), the selected independent formulation and process variables were: PLGA amount (X1), liraglutide amount (X2), 2nd sonication time (X3), PVA concentration (X4), lyoprotectant type (X5), lyoprotectant concentration (X6) and external aqueous phase to organic phase w2/o ratio (X7). Particle size (Y1), PDI (Y2), EE (Y3) and zeta potential (Y4) were selected as dependent variables. The examined lower and upper levels of the independent factors X_1_-X_7_ (Table 1) were also determined depending on preliminary experiments and literature survey.. The design was validated by 3 extra center checkpoint formulations and the bias (%) between predicted and observed values of response was calculated. The optimized formulation was prepared within design space (DS) and compared with predicted results of the responses.

**Table 1:** Levels of the Selected Critical Factors

Critical factor		Low level	High level
PLGA amount (mg)	X1	30	60
Liraglutide amount (mg)	X2	0.5	5
2nd sonication time (min)	X3	0.5	2
PVA (%)	X4	0.5	2
Lyoprotectant type	X5	Mannitol	Trehalose
Lyoprotectant (%)	X6	5	10
W2/O ratio	X7	2	5

#### Characterization of the Prepared Liraglutide Loaded PLGA NPs

##### Particle Size, Size Distribution and Surface Charge Measurements

Approximately 5 mg of the prepared freezedried NPs was dispersed in 5 ml of double distilled water and sonicated to minimize the possible inter-particle interactions. The hydrodynamic diameter (Z-average), PDI and zeta potential of reconstituted NPs were measured in folded capillary cell by using Malvern Nano ZS Zetasizer (Malvern Instruments Ltd. UK) equipped with He-Ne laser(633 nm). The instrument allows the particle size measurement in the range of 0.3 nm-10.0 μm using patented *NIBS* (Non-Invasive Back Scatter) technology, with high accuracy of ± 2%. The samples were measured at 25°C, the refractive index was 1.445, and the number of scans was 17. All the measurements were conducted in triplicate and the average value of each was used.

##### Encapsulation Efficiency (EE)

The encapsulation efficiency of liraglutide encapsulated in PLGA NPs was determined directly using the centrifugation method. In this method, 20 mg from each NPs formulation was dissolved in 2 ml of DCM, then liraglutide was extracted into 4 ml of PBS (pH = 8.1), soaked for 30 min and then centrifuged at 16500 rpm at 4^ο^C for 15 min. The supernatant was then collected and the amount of encapsulated liraglutide in the supernatant was measured using the RP-HPLC method. Samples were run in triplicate.

The percentage of EE was calculated using the following equations:

$$ \mathrm{EE}\%=\mathrm{encapsulated}\ \mathrm{amount}\ \mathrm{of}\ \mathrm{liraglutide}/\mathrm{total}\ \mathrm{amount}\ \mathrm{of}\ {\mathrm{liraglutide}\ \mathrm{added}}^{\ast }\ 100. $$

##### Chromatographic Equipment and Conditions

Reversed phase HPLC (Shimadzu Corporation, NEXERA X2, Tokyo, Japan) method was developed in our lab to analyze liraglutide. A Kinetex ® C18 column with dimensions of (5 μm, 150*4.6 mm, (Phenomenex, USA) was used as a stationary phase. The flow rate of 1.5 ml/min was set over 15 min with a mobile phase comprised of 0.02 M aqueous KH2PO4 solution (pH = 7.0, solvent A) and acetonitrile (solvent B). The mobile phase was pumped in a gradient mode as it was changed from 80:20 (A:B, *v*/v) to 30:70 (A:B, v/v) in 12 min then going back to 80:20 (v/v) between 12.1–15 min. The column temperature was set to 40°C, and the sample tray temperature was set to 15°C. Fifty microliters of sample volume was injected. The wavelength of UV detection was 214 nm.. The retention time of liraglutide is 8.65 min. In our HPLC method, some chromatographic parameters have been calculated. The limit of detection (LOD) value of the liraglutide is 0.175 ppm, the limit of quantification (LOQ) value is 0,530 ppm, respectively. Capacity factor (k’) for liraglutide is 5.20, the asymmetry factor of the peak of liraglutide showed 1.40 value, respectively. The theoretical plate (N) value is 146,620, calculated by the Ph.Eur. guideline. The regression of the linearity (R^2^) of the liraglutide calibration curve was 0.996, respectively.

##### Scanning Electron Microscopy Measurements (SEM)

To investigate the surface morphology, sphericity, and discreteness of the freeze dried NPs containing lira, scanning electron microscopy (SEM) (Hitachi S4700, Hitachi Scientific Ltd., Tokyo, Japan) at 10 kV was used. The samples were coated with gold-palladium (90 s) with a sputter coater (Bio-Rad SC 502, VG Microtech, Uckfield, UK) using an electric potential of 2.0 kV at 10 mA for 10 min. The air pressure was 1.3–13.0 mPa. 3 repetitions of the optimized formula were tested by SEM technique and images were captured from different surface regions of each sample and at two different magnifications (×15,000, ×45,000).

#### Compatibility Studies

To investigate the physicochemical compatibility between the drug and the polymer in the prepared PLGA NPs, FTIR, DSC and XRD analysis were conducted.

##### Fourier Transform Infrared Spectroscopy (FTIR)

The FT-IR spectra of pure Lira, PLGA, Lira free/loaded PLGA NPs were recorded using FT-IR spectrometer (Thermo Nicolet AVATAR; LabX Midland, ON, Canada) in the range of 4000 and 400 cm^−1^ with an optical resolution of 4 cm^−1^. The sample was mixed with 150 mg of dry KBr and compressed to prepare the pellet.

##### Differential Scanning Calorimetry (DSC)

To define the physical state of the peptide drug in the nanoparticles and assess any possible intermolecular interaction between the drug and the polymer in the nanoparticles, DSC studies of pure Lira, PLGA, Lira free/loaded PLGA NPs were performed using (Mettler Toledo TG 821e DSC Mettler Inc., Schwerzenbach, Switzerland). Accurately weighed samples (3–5 mg) were sealed in an aluminum pan and an empty pan was used as a reference. The samples were analyzed at a scanning temperature from 25 to 300°C at a heating rate of 10°C/min under nitrogen purge. Data analysis was performed using the STAR^e^ software (Mettler Toledo Mettler Inc., Schwerzenbach, Switzerland).

##### X-Ray Diffraction Study (XRD)

XRD is a useful technique applied herein to characterize the physical state of liraglutide entrapped in PLGA NPs and further to confirm the stability attributed to polymer-drug interaction. Powder X-ray diffraction (XRD) patterns of pure Lira, PLGA, Lira free/loaded PLGA NPs were obtained using an X-ray powder diffraction (XRPD) BRUKER D8 Advance X-ray diffractometer (Bruker AXS GmbH, Karlsruhe, Germany), supplied with a Cu K λ1 radiation source (λ = 1.54056 A°), with a voltage of 40 kV and a current of 40 mA, in flat plate θ/2θ geometry, over the 2θ ranges 3–40°, with a scan time of 0.1 s at step size of 0.007°. The sample was placed on a quartz holder and measured at ambient temperature and humidity.

#### Stability of Lira Encapsulated in PLGA NPs

##### Electrospray Ionization Mass Spectrometry

Electrospray Ionization Mass Spectrometry (ESI-MS) is a valuable tool to be used to provide information about the molecular weight of native Lira and compare it to Lira loaded in NPs. Lira was characterized by an Agilent 1100 LC-MSD trap mass spectrometer equipped with an electrospray ion source.

##### Circular Dichroism

CD was performed to evaluate the conformational stability of Lira loaded into the prepared polymeric NPs. CD spectra were obtained with a Jasco J-1100 spectropolarimeter (Tokyo, Japan). Aliquot of each of PBS (pH = 8.1), native Lira in PBS and Lira extracted from NPs in PBS was placed in a 10 mm pathway Far-UV quartz cuvette and the Far-UV CD spectra were collected by an PM-539 CD spectrometer. Spectra were collected at room temperature over the wavelength range of 260 nm to 195 nm with 0.2 nm interval. Ellipticity was recorded at scanning speed of 100 nm/min and 1.00 nm band with 5 accumulations. PBS solution subtraction, noise reduction and data analysis were performed using standard analysis and temperature/wavelength analysis programs (Jasco).

## Results and Discussion

### Placket Burman Design: Risk Analysis

The QTPP that encompasses the desired CQAs was defined in our previous paper as following: stable, homogeneous and spherical shaped freeze dried NPs with particle size of 100-300 nm and maximum EE. Risk assessment was also conducted (using LEAN-QbD software) for ranking and prioritizing CMAs and CPPs likely to have an impact on the quality of Lira loaded PLGA NPs (20) and the highly influential parameters were prioritized and subjected to subsequent screening using a seven-factor, two-level, eight-run PB screening design (Table 2) in order to minimize their risk to a low level by controlling these variables in a specific accepted range.

**Table 2:** The Input Factor Levels in 7 Factor, 2 Level, 8 Run PBD

Run code	PLGA (mg)	Lira (mg)	2nd sonication time (min)	PVA (%)	Lyoprotectant type	Lyoprotectant (%)	W_2_/O ratio
PBD-F1	30	0.5	0.5	2	Trehalose	10	2
PBD-F2	60	0.5	0.5	0.5	Mannitol	10	5
PBD-F3	30	5	0.5	0.5	Trehalose	5	5
PBD-F4	60	5	0.5	2	Mannitol	5	2
PBD-F5	30	0.5	2	2	Mannitol	5	5
PBD-F6	60	0.5	2	0.5	Trehalose	5	2
PBD-F7	30	5	2	0.5	Mannitol	10	2
PBD-F8	60	5	2	2	Trehalose	10	5

Herein, the possible effects of these formulation and process parameters on four responses namely: mean particle size, PDI, EE and zeta potential were investigated by applying the PB screening design where the experimental data were validated by ANOVA for each factor. ANOVA parameters for predicting mean particle size (Y1), PDI (Y2), EE (Y3) and Zeta potential (Y4) are presented in Supplemental Table 1.Surface response plots are also useful diagrammatic representation of the values of the response, to project the significance of effects for each variable and can explain the relationship between tested independent factors and dependent responses. A color-scale object along with the surface plot serves as a legend, and the value of the response is dependent on the gradual color. Based on this color gradient, these plots can present the change in response value with different levels of the independent variable.

### Influence of Investigated Parameters on the Z-Average Size, PDI, EE and Zeta Potential

Depending on the selected parameters levels, the Z-average ranged between 160.1 ± 5.6 nm and 235. ± 5.3 (Table 3). The corresponding coefficients are summarized in Supplemental Table 2, where the factors a *P* value<0.05 are regarded as highly significant, while the ones having nonsignificant response coefficients with a P value>0.05 are least contributing in the prediction of mean particle size. The polynomial equation obtained for the fitted full model explaining the effect of formulation and process variables on the mean particle size is:

**Table 3:** Experimental Responses Results in PBD

Run code	Z-AVE (nm)	PDI	EE%	Z-potential (mV)
PBD-F1	160.1 ± 5.6	0.10 ± 0.03	20.1 ± 1.7	−30.6 ± 1.8
PBD-F2	209.8 ± 8.01	0.15 ± 0.01	36 ± 1.25	−25 ± 1.6
PBD-F3	190.0 ± 2.8	0.23 ± 0.01	41 ± 2.46	−27.3 ± 0.7
PBD-F4	200.2 ± 3.5	0.16 ± 0.03	43.5 ± 3.34	−31.2 ± 1.2
PBD-F5	179.5 ± 3.8	0.17 ± 0.01	32 ± 4.03	−23.8 ± 1
PBD-F6	235.7 ± 5.3	0.10 ± 0.003	28.9 ± 2.05	−29.2 ± 0.4
PBD-F7	223.6 ± 3.7	0.17 ± 0.02	22.2 ± 2.12	−30.4 ± 0.
PBD-F8	183.5 ± 3.03	0.20 ± 0.01	21 ± 1.51	−26 ± 0.2

$$ {\displaystyle \begin{array}{l}{\mathrm{Y}}_{1=}197{.8021}_{+}9.5079{\mathrm{X}}_1+1.5304{\mathrm{X}}_2+7.7638{\mathrm{X}}_3-16.9754{\mathrm{X}}_{4-}5.4712{\mathrm{X}}_5-3.5429{\mathrm{X}}_6-7.0791{\mathrm{X}}_7\mathrm{with}\ {\mathrm{R}}^2=0.9745,\\ {}\mathrm{adjusted}\ {\mathrm{R}}^2=0.9609,\mathrm{and}\ \mathrm{Mean}\ \mathrm{square}\ \left(\mathrm{MS}\right)=22.3978\end{array}} $$

**Fig. 1:**
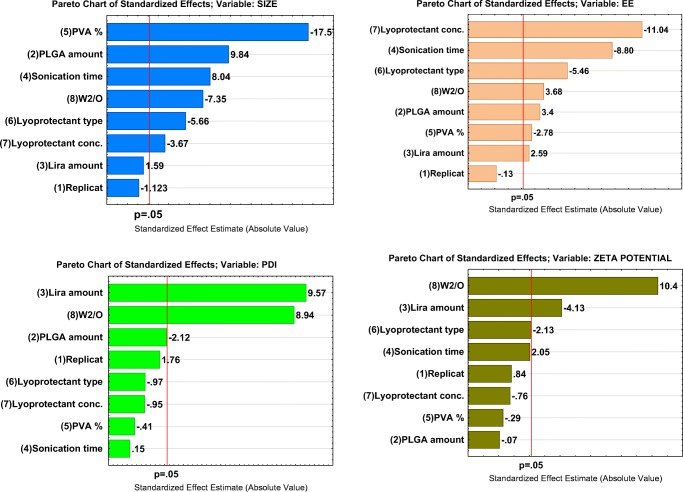
Pareto charts of the effects of the examined independent variables on Z-average size (Y1), PDI (Y2), EE (Y3), zeta potential (Y4). Replicate refers to number of repetitions for each formula which was 3.

According to this polynominal equation and the Pareto chart (Fig. 1), the most influential factors in terms of particle size are the PVA concentration, PLGA amount followed by sonication time and W2/O ratio at almost the same level of significance. Then the next important variables include lyoprotectant type and concentration. The tested levels of Lira amount were observed to have a non-statistically significant effect on the mean particle size.

In all prepared formulations, NPs exhibited a practically monodisperse or narrow distribution (21) as PDI was ranged from 0.1 ± 0.003 in PBD-F6 to 0.22 ± 0.01 in PBD-F3 (Table 3) evidencing that the obtained NPs are homogeneous and stable with no aggregation. Figure S1 shows an example of PDI = 0.07 with Z-average = 157.1 nm obtained for PBD-F1. The polynomial equation obtained for the full model describing the effect of formulation and process variables on the PDI value is:

$$ {\displaystyle \begin{array}{l}\mathrm{Y}2=0.1568-0.0061\mathrm{X}1+0.0312\mathrm{X}2+0.0005\mathrm{X}3-0.0013\mathrm{X}4-0.0032\mathrm{X}5-0.0031\mathrm{X}6+0.0291\mathrm{X}7\\ {}\;\mathrm{with}\ {\mathrm{R}}^2=0.9235,\mathrm{adjusted}\ {\mathrm{R}}^2=0.8828,\mathrm{and}\ \mathrm{Mean}\ \mathrm{square}\ \left(\mathrm{MS}\right)=0.0003\;\\ {}\mathrm{Y}2=0.1568-0.0061\mathrm{X}1+0.0312\mathrm{X}2-0.0032\mathrm{X}5-0.0031\mathrm{X}6+0.0291\mathrm{X}\ 7\dots \dots \dots \mathrm{Redcuced}\left(\mathrm{model}\right)\\ {}\;\mathrm{with}\ {\mathrm{R}}^2=0.92256,\mathrm{adjusted}\ {\mathrm{R}}^2=0.89522,\mathrm{and}\mathrm{Mean}\ \mathrm{square}\ \left(\mathrm{MS}\right)=0.0002.\end{array}} $$

The statistical analysis (Table 3S) along with the Pareto chart (Fig. 3) revealed that only two of the examined CMAs namely Lira amount and W_2_/O ratio were observed to have a significant effect on the PDI value.

Depending on the tested two levels of each factor, the results showed that EE varied between 20.1 ± 1.7 in formulation PBD-F1 and 43.5 ± 3.3in PBD-F4 (Table 3).The polynomial equation obtained for the fitted full model showing the impact of the seven examined variables on EE is:

$$ {\displaystyle \begin{array}{l}\mathrm{Y}3=30.548+1.7683\ {\mathrm{X}}_1+1.3458{\mathrm{X}}_2-4.5825{\mathrm{X}}_3-1.4458{\mathrm{X}}_4-2.8408{\mathrm{X}}_5-5.7483{\mathrm{X}}_6+1.91{5\mathrm{X}}_7\;\\ {}\mathrm{with}\ {\mathrm{R}}^{2=}0.9471,\mathrm{adjusted}\ {\mathrm{R}}^2=0.9189,\mathrm{and}\ \mathrm{Mean}\ \mathrm{square}\ \left(\mathrm{MS}\right)=6.5021.\end{array}} $$

This equation along with the Pareto chart (Fig. 1) and statistical analysis (Table 4S) show that the lyoprotectant %, 2nd sonication time and lyoprotectant type are the most highly risky factors in terms of EE. This is followed by other factors which all show a significant impact on the amount of Lira encapsulated in the PLGA NPs.

The zeta potential was also monitored during the optimization steps and its values ranged from-31.2 ± 1.2 mV in PBD-F4 to −23.8 ± 0.95 mV in PBD-F5 (Table 3), and these expected negative values are attributed to the presence of carboxyl group end on PLGA. Figure S2 depicts the result of zeta potential obtained for formulation PBD-F1 as an example. The full model describing the effect of formulation and process variables on the zeta potential is:

$$ {\displaystyle \begin{array}{l}{\mathrm{Y}}_{4=}-28.0596-0.0154{\mathrm{X}}_1-0.9071{\mathrm{X}}_2+0.4496{\mathrm{X}}_3-0.0646{\mathrm{X}}_4-0.4679{\mathrm{X}}_5-0.1663{\mathrm{X}}_6+2.2846{\mathrm{X}}_7\mathrm{with}\ \mathrm{R}2=0.9001,\\ {}\;\mathrm{adjusted}\ \mathrm{R}2=0.8468,\mathrm{and}\ \mathrm{Mean}\ \mathrm{square}\ \left(\mathrm{MS}\right)=1.1599\\ {}\mathrm{Y}4=-28.0596-0.9071\mathrm{X}2+0.4496\mathrm{X}3-0.4679\mathrm{X}5-0.1663\mathrm{X}6\\ {}+2.2846\mathrm{X}7\dots \dots \dots \dots \mathrm{R}\mathrm{educed}\ \left(\mathrm{model}\right)\ \mathrm{with}\ \mathrm{R}2=0.89946,\mathrm{adjusted}\ \mathrm{R}2=0.86397,\mathrm{and}\ \mathrm{Mean}\ \\ {}\;\mathrm{square}\ \left(\mathrm{MS}\right)=\mathrm{1.0.297}.\end{array}} $$

It is obvious from the statistical analysis (Table 5S) and the Pareto chart (Fig. 1) that only the W2/O ratio and the lira amount had a significant impact (*P* < 0.05) on the surface charge of PLGA NPs. Other examined variables were observed to have only a non-significant effect on surface charge.

The effect of the above-explained variables on Y1,Y2,Y3 and Y4 is discussed point by point in the following:

#### Effect of Polymer Amount

**Fig. 2:**
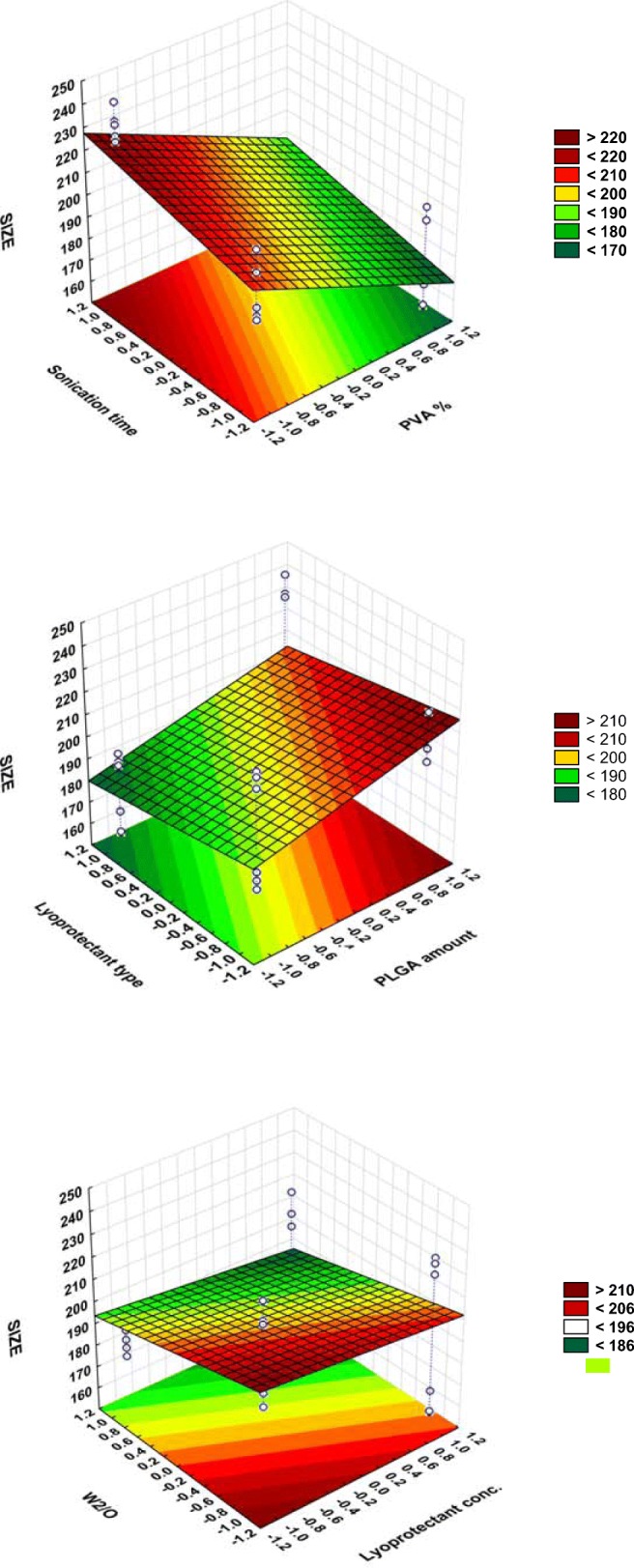
Surface plots showing the effect of the significant examined variables on the Z-average size (Y1).

It is apparent from Fig. 2 that when the PLGA amount was increased, the Z-average increased correspondingly, as supported by many earlier published papers (22,23) That could be explained by increasing the viscosity of the organic phase which leads to a reduction in the net shear stress (24), in addition to a reduction in the evaporation rate; i.e. the dispersion rate of the organic phase toward the external aqueous phase will be slower, thus that incites the formation of larger particles (25,26). The formation of a more viscous organic phase was reported to push up the frequency of collisions between particles during the emulsification and droplet solidification step, which may lead to the aggregation of the semisolid particles (5). Regarding the PDI value, it was also observed that size distribution was slightly decreased by increasing the PLGA amount which means that a greater level of PLGA would promote the formation of much more homogeneous NPs.

The positive effect that the PLGA amount has on EE (Fig. 4) could be again due to the fact of increasing the viscosity of the organic phase with a higher amount of polymer, which can retard drug diffusion into the external aqueous phase and thus increase the amount of drug entrapped inside the NPs (27,28). It is also known that larger nanoparticles obtained with a higher PLGA level can provide sufficient surface for entrapping the peptide drug. Furthermore, higher PLGA levels give rise to more rapid polymer deposition as ethyl acetate is removed from the NPs, which is expected to hinder any undesirable Lira diffusion into the external phase (29).

#### Effect of Liraglutide Amount

**Fig. 3:**
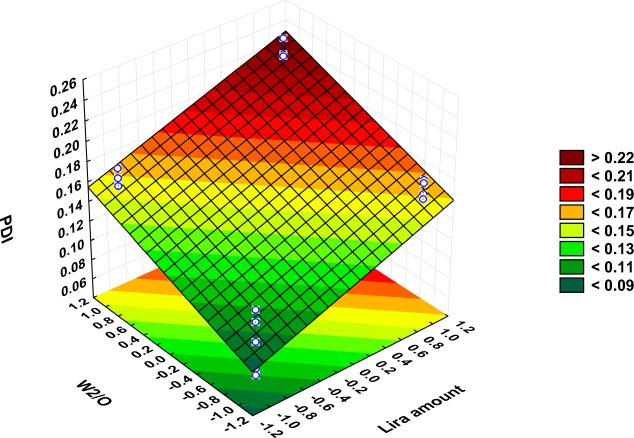
Surface plot showing the effect of lira amount and W_2_/O ratio on PDI (Y2).

**Fig. 4:**
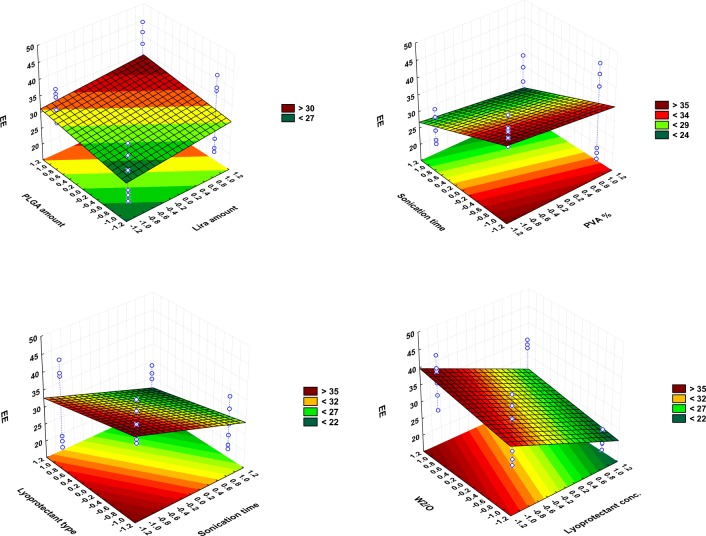
Surface plot showing the effect of examined variables on EE (Y3).

**Fig. 5:**
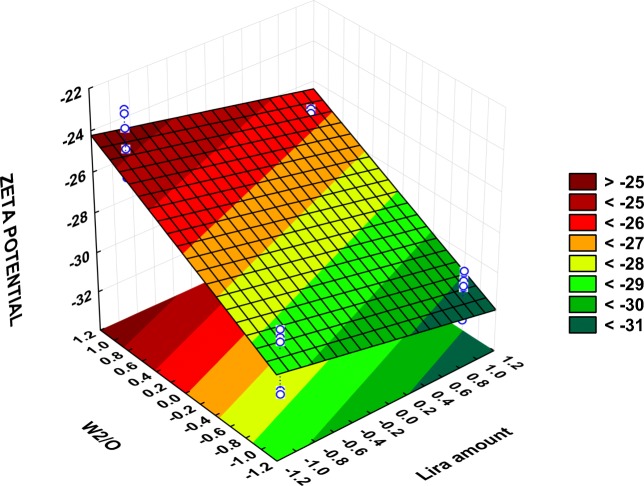
Surface plot showing the effect of lira amount and W_2_/O ratio on zeta potential (Y3).

The positive effect that Lira has on the 2nd emulsification and both final particle size (Fig. 2) and size distribution as a result (Fig. 3) would be explained by the influence of the drug on the droplet size of the inner aqueous phase within the organic phase in the first water-in-oil (w1/o) emulsion, which may modify its ability for dispersion in the outer aqueous phase. However, this effect was only limited and not significant in the case of particle size, while Lira theoretical loading amount was a significant influential parameter affecting the PDI and zeta potential values (Fig. 1, Fig. 3, Fig. 5). The Lira level was also shown to have a significant positive effect on EE; when loading higher amount of Lira, EE was even higher (Fig. 4). This positive trend was previously reported with other peptide drugs as insulin, and it is explained due to higher amount of peptide that is associated with the surface of nanoparticles and is electrooptically linked to a greater extent (30), thus resulting in a higher EE value. However, the studied levels of the Lira amount in our work were shown to be the least risky factor affecting EE as presented in the Pareto chart (Fig. 1).

#### Effect of PVA Concentration

PVA has previously proved to be a good choice as a surfactant used to prepare stable PLGA NPs with a small size and a narrow PDI due to its ability of minimizing the surface tension of the continuous phase which is an aqueous phase in our work. The statistical analysis of the results showed that increasing PVA level played a crucial role in decreasing particle size (Fig. 2), and it was the leading factor impacting the size of NPs as can be seen in the Pareto chart (Fig. 1) which is in agreement with previous reports (31,32) .This result can be expected from the stabilizing function of PVA molecules that tend to align themselves at the droplet surface lowering the free energy at the interface between two phases and avoiding coalescence between nanodroplets, thus stabilizing the smaller droplets and preventing coalescence into a larger one (33,34). Hence, at a low PVA concentration, a larger particle size was obtained due to insufficient reduction in interfacial tension. It was also reported that a fraction of PVA remains associated with the surface of nanoparticles even after the washing of nanoparticles (33). Thus, the presence of the PVA layer on the surface of nanoparticles may also improve their stability during the freeze-drying process.

It is also clear that as PVA level increased from 0.5% to 2%, EE decreased (Fig. 4). A possible explanation of this negative impact was discussed in a preceding published work, as it was proved that the breakdown of the inner aqueous droplets containing Lira took place along with the fragmentation of the organic phase because of the cavitation occurs in the complex system of three phases, the higher level of PVA in the external aqueous phase is attributed to enhancing the breakdown of inner aqueous droplets and a higher amount of Lira can escape to the external phase as a result (34). This is supported by a previous paper in which increasing the emulsifier concentration had led to lower entrapment of the protein drug in PLGA NPs, which was explained as a result of increasing the partitioning of the drug from the inner to the outer phase (35).

#### Effect of 2nd Sonication Time

The results revealed that larger and less homogeneous particles were yielded when increasing 2nd sonication time from 30 s to 2 min (Fig. 1, Fig. 2). This could be explained as follows: at the beginning, increasing the sonication time led to the formation of smaller droplets due to the production of higher energy and higher shearing rates, which are more efficient in breaking large droplets into smaller ones (36). However, a further elevation in this sonication period resulted in the re-aggregation of these particles. This trend is in accordance with results obtained by others where they observed the formation of larger droplets as an outcome of longer sonication or homogenization time (37). Besides, the longer the 2nd sonication time, the higher the shear energy input, thus the higher the leached amount of peptide from W1/O to the external aqueous phase i.e. the lower the EE. The Pareto chart (Fig. 1) demonstrates that the prolonged 2nd sonication time was the 2nd highest risky factor regarding the influence on EE.

#### Effect of Lyoprotectant Type and Concentration

Lyoprotectant are commonly used to stabilize the particles and protect them from degradation during freeze-drying and storage (38). Regarding the type of lyoprotectant used in this study, these significant changes in particle size may be related with the behavior of each lyoprotectant during freeze-drying, and the adsorption of lyoprotectant on the surface of nanoparticles. It is clear that trehalose is more effective in obtaining smaller nanoparticles (Fig. 1, Fig. 2).This is in accordance with previous papers that confirmed that trehalose which is a non-reducing sugar could be the most preferable lyoprotectant of choice because of its merits over the other sugars; including a very low chemical reactivity, a higher glass transition temperature Tg, less hygroscopicity, in addition to the absence of internal hydrogen bounds, allowing a more flexible formation of hydrogen bonds with nanoparticles during the freeze-drying process (39). However, mannitol was proved to be more effective in obtaining a higher EE value according to our experimental work. Trehalose was investigated before regarding its effect on the secondary structure of insulin, and the results showed that it highly affected the conformational stability of the peptide; so it might not be the best choice to encapsulate peptide drugs (40). In addition to that; mannitol is able to form crystal morphology (which is confirmed later in this paper by DSC and XRD) and this might be attributed to the stability of peptide.

When it comes to the lyoprotectant level, results revealed that increasing this level up to 10% significantly reduced the Z-average and slightly minimized the PDI of the obtained NPs (Fig. 1,Fig. 2), which means that at this level the used lyoprotectants are more efficient in preventing the aggregation and stabilizing the PLGA NPs the use of an excess amount of lyoprotectant might eventually make it reach the limit of its stabilization ability and thus the agglomeration of NPs is likely to increase (41). It is also apparent from the statistical analysis presented in the Pareto chart and surface plot (Fig. 1, Fig. 4) that the lyoprotectant level was the most influential formulation variable impacting EE. When the level of lyoprotectant continued increasing, the amount of entrapped Lira significantly decreased, which could be the result of smaller NPs obtained with a higher lyoprotectant level, and thus less sufficient surface area for entrapping the drug.

#### Effect of W_2_/O Ratio

As the volume ratio of external aqueous phase to organic phase W2/O went on increasing, which was achieved by increasing the volume of the external aqueous phase, the average particle size was significantly decreased as can be seen from the Pareto chart and surface plot (Fig. 1, Fig. 2). This formation of smaller droplets may be due to the higher amount of stabilizer present as compared to the non-sufficient amount of stabilizer when using a lower amount of this phase. It was also recently reported that increasing the continuous phase volume:organic phase ratio had led to particle size reduction (27).

Regarding PDI values, a significant increase in size distribution was observed when the volume of the external aqueous phase was higher, as shown by the Pareto chart and surface plot (Fig. 1, Fig. 3). This observation might be attributed to a reduction of shear stress during the homogenization process (42). Besides, the phase ratio was the highly influential factor affecting the surface charge as increasing the external aqueous phase volume led to a significant increase in the zeta potential value (Fig. 5).

The EE exhibited a significant upward trend when increasing the volume of the external aqueous phase, as presented in the Pareto chart and surface plot (Fig. 1,Fig. 4). The impact of the external aqueous phase/organic phase is controversial, as many papers reported that increasing the W2/O ratio can lead to minimizing the amount of the encapsulated drug (31,42). However, other published papers assumed that a relatively higher volume ratio of the external aqueous phase was beneficial for maximizing the drug encapsulated in the NPs as a higher outer aqueous phase volume can speed up the solidification time (evaporation of ethyl acetate and formation of NPs), while the smaller the volume of this outer aqueous phase, the longer the time required for solidification, thus over this time Lira may leak to the outer phase due to its hydrophilicity (43).

### Placket Burman Design: Model Validation

The three replications of center checkpoint formulations were prepared and evaluated for the particle size, EE, PDI and zeta potential to evaluate the reproducibility of the generated models and estimate the experimental error. Table 4 presents the percentage of bias between predicted and observed values for each response was calculated by means of the following equation .

$$ \mathrm{Bias}\ \left(\%\right)=\left(\mathrm{Predicted}\ \mathrm{value}-\mathrm{observed}\ \mathrm{value}\right)/\mathrm{Predicted}\ {\mathrm{value}}^{\ast }100 $$

**Table 4:**
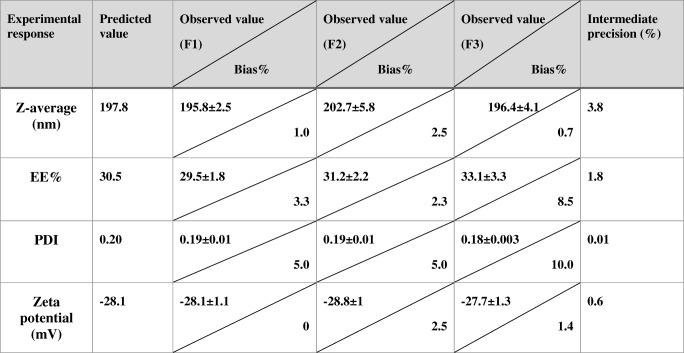
The Observed and the Predicted Values of the Response Values of the Center Checkpoints

The minor differences between the predicted values and the average of experimental values confirm the validity of this design in providing a good prediction of the four tested responses.

In addition to that, the calculated relative standard deviation RSD% values that are presented in Table 4 prove the repeatability and intermediate precision regarding the 4 responses that further confirms high analytical process variability.

### Placket Burman Design: Design Space and Optimization

**Fig. 6:**
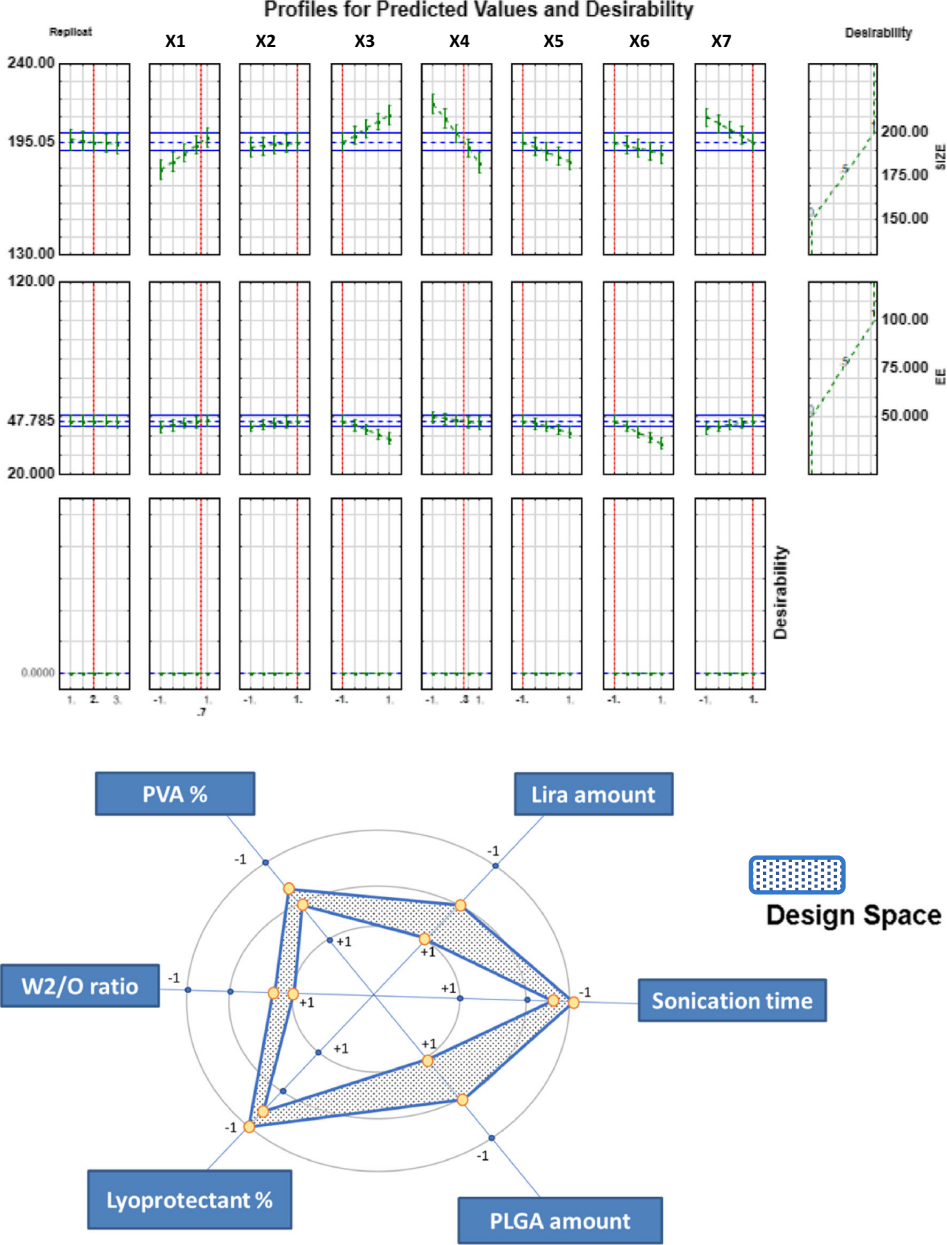
The desirability plots and graphical design space representing the optimum levels of factors required to prepare the optimized formula.

After establishing the polynomial equations describing the relationship between the CPPs, CMAs and the examined responses namely; particle size, EE, PDI and zeta potential, the optimization process was conducted. Among the four responses, size and EE were the highly critical quality attributes of nanoparticles being significantly affected by almost all the tested variables which is in accordance with the estimated severity scores of CQAs that was calculated previously at the initial risk assessment process. Therefore, the deign space (DS) was optimized (Fig. 6) targeting the following criteria: the particle size was minimized, encapsulation efficiency was maximized while PDI and Zeta potential were excluded. Thanks to the knowledge obtained via the DS, the optimum levels of the formulation factors were determined: 60 mg of PLGA, 5 mg of Lira, 0.5 min 2nd sonication time, 1.48% of PVA, 5% of mannitol and W2/O ratio of 5.As shown in Table 5, the observed values were comparable to the predicted ones, presenting another confirmation of the validity of the generated models and indicating that the optimized formulation is reliable.

**Table 5:** The Observed and the Predicted Values of the response values of the Optimum Lira Nanoparticle

Experimental response	Predicted value	Observed value	Residual	Bias (%)
Z-average (nm)	197.9	189 ± 4.99	8.95	4.5
EE%	48.3	51.8 ± 2.39	3.5	7.2
PDI	0.21	0.19 ± 0.012	0.034	7.8
Zeta potential (mV)	−26.5	−27.1 ± 1.33	0.58	2.2

### Scanning Electron Microscopy (SEM)

**Fig. 7:**
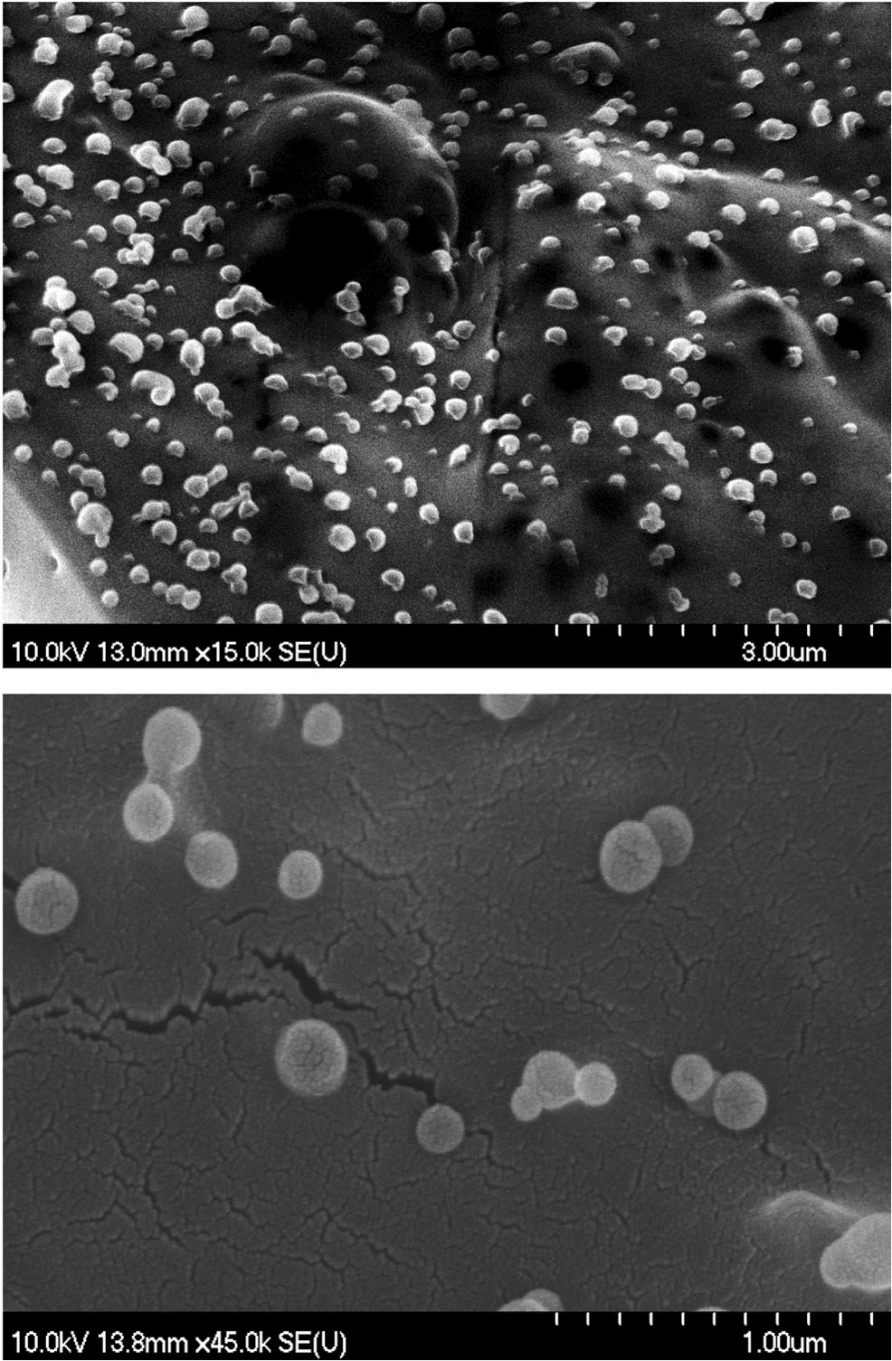
SEM images of liraglutide loaded PLGA NPs.

Figure 7 depicts the shape and surface morphology of the optimized Lira loaded PLGA NPs visualized by SEM. Since the optimized formula was homogeneous (in accordance with low PDI) we selected two images as representative for the sample. The results revealed that Lira loaded PLGA NPs were spherical with quite a smooth surface and they had homogeneous distribution which is in agreement with the above-mentioned results that demonstrated low PDI values for all formulations.

### Compatibility Studies

#### Fourier Transform Infrared Spectroscopy (FTIR)

Figure S3. represents the FTIR spectra of pure Lira, pure PLGA and Lira free/loaded PLGA NPs.

The amide I region (1710–1590 cm^−1^) is the most representative region of the spectra to assess peptide or protein based drug secondary structure (44). In the FTIR of pure Lira, the amide I band was located at 1655 cm^−1^ and was assigned to C=O stretching, while the amide II band was observed at 1541 cm^−1^ (in-plane N–H bending component and C–N stretching bands of the amide bond). Besides, the typical peak at 2928 cm^−1^ was ascribed to C-H stretching of CH3, and the peak at 1396 cm^−1^ is attributed to amide III. When analyzing the FTIR spectra of Lira loaded PLGA NPs, it was found that the major peaks of pure Lira assigned to amide I and II and III were masked by the PLGA bands, and this seems to be a logical result since the amount of PLGA was much higher than the Lira amount in these NPs. There were no clear differences between the spectra of the blank NPs and Lira loaded NPs which is also expected as the drug loading is very small when compared to the polymer amount. These observations suggest that Lira was successfully loaded into the PLGA NPs.

#### Differential Scanning Calorimetry (DSC)

Figure S4 represents the DSC thermograms for pure Lira, PLGA, liraglutide free/loaded PLGA NPs. For the temperature range examined, the PLGA thermogram exhibited a glass transition point at 49.22°C and no melting endothermic peak was observed, as PLGA appears amorphous in nature. The DSC thermogram of pure Lira revealed a peak at 275.46°C, which is attributed to the thermal degradation of this peptide drug, and no endothermic peak of melting was shown, which proved the amorphous nature of the drug. Since the thermogram of Lira loaded PLGA NPs did not display any extra endo/exothermic peaks compared to the blank NPs, this is an indicator of the presence of Lira in the amorphous phase and this drug is successfully encapsulated into the PLGA matrix.

#### X-Ray Diffraction Study (XRD)

XRD studies further verified the amorphous nature of both PLGA and pure Lira as they showed no characteristic peaks in their diffractograms which is in accordance with the results of DSC thermograms (Fig. S4). As depicted in Fig. S5, mannitol remained in crystalline state after freeze drying which is due to the property of mannitol to recrystallize at low cooling rates rather than rapid cooling. The crystallization of this lyoprotectant could have a negative effect on the stability of NPs as it is able to limit the formation of these hydrogen bonds (30), and this can explain why trehalose was more efficient than mannitol at preventing the aggregation of NPs and thus minimizing the Z-average. There was no difference between the diffractograms of the loaded and blank PLGA NPs which is explained in literature as a result of the successful encapsulation of the peptide drug inside the polymeric nanoparticles without change in its physical state (45), and this is in accordance with the DSC results.

### Structural Stability

#### ESI-MS

**Fig. 8:**
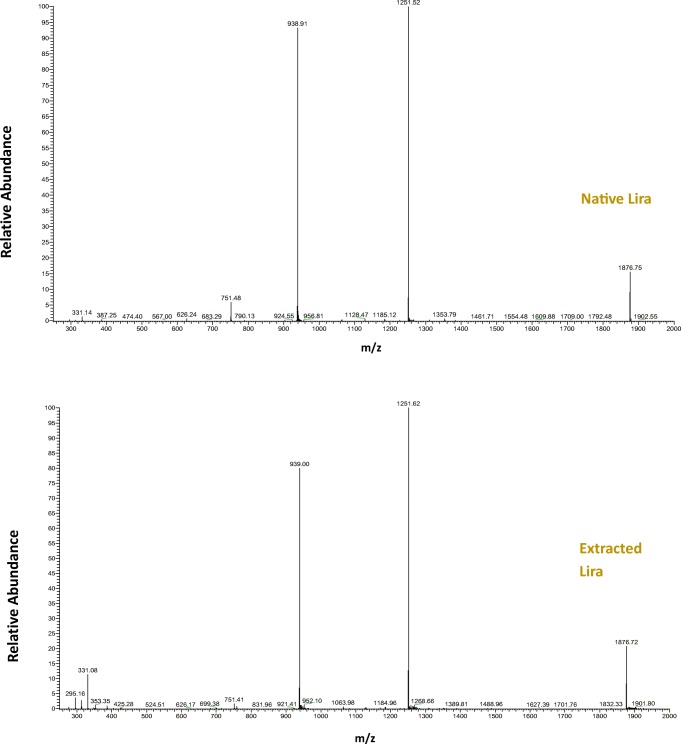
Mass spectra of lira extracted from PLGA NPs compared to native lira.

MS was used to compare the molecular weight MW of lira standard (native) to lira extracted from PLGA NPs. As the spectra in Fig. 8 depicts, the measured MW of native Lira and Lira loaded in NPs are almost equal, and the spectra confirmed the presence of Lira with a molar mass of 3751 Da which is an evidence of the integrity of Lira loaded in PLGA NPs prepared using the optimized formulation and process parameters.

#### CD

**Fig. 9:**
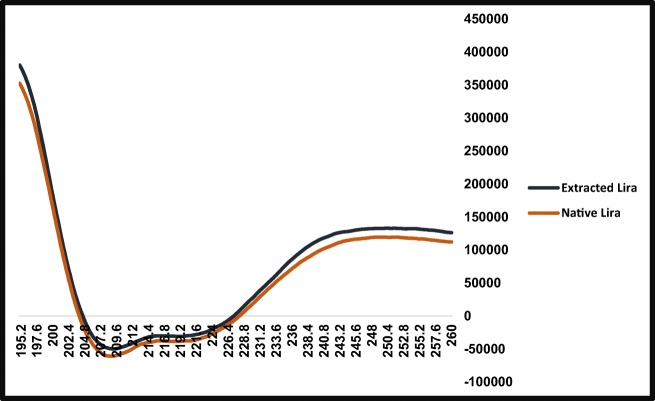
CD spectra of lira extracted from PLGA NPs compared to native lira.

Since the preservation of the secondary structural integrity of a peptide drug in the nanocarrier is critical for its biological efficacy, the secondary structure of Lira extracted from NPs was compared to that of native Lira. The CD spectra of native Lira (Fig. 9) showed two minima at 208.8 nm and 218.4 nm indicating the presence of alpha helix elements in the structure, which is in consistent with previous studies on the typical structure of the glucagon-like peptide-1 family. No significant conformational change was recorded for Lira extracted from PLGA NPs (in PBS, pH = 8.1) as the far UV CD spectra for it showed two minima at 209.4 nm and 219.2 nm, and almost entirely overlapped with the CD spectrum for the standard.

## Conclusion and Future Perspectives

The present study is the first published work that substantiated the application of rational QbD-based methodology for the optimization of a GLP-1 analog loaded nanocarrier system. This work demonstrated the importance of implementing DOE within QbD philosophy in the early stage of Liraglutide containing NPs development due to the complexity of this system.

After establishing the design space, with the minimum particle size and maximum EE, the optimized formula was successfully prepared meeting the targeted CQAs. This optimized Lira loaded PLGA NPs formula was also successful in maintaining the native structure of Lira and could be promising for the oral delivery. Thus, *in vitro* release kinetics, cytotoxicity, intestinal permeability and *in vivo* studies will be further conducted on this formula.

## Electronic supplementary material

ESM 1 (DOCX 1133 kb)

ESM 2 (DOCX 24 kb)
