# Supplementary material for: Synthesis and Statistical Optimization of Poly (Lactic-Co-Glycolic Acid) Nanoparticles Encapsulating GLP1 Analog Designed for Oral Delivery
Source: Pharm Res. 2019 May 13;36(7):99. doi: 10.1007/s11095-019-2620-9 (PMC6513835; doi:10.1007/s11095-019-2620-9)
Supplement: Supplementary file 1 — (DOCX 1133 kb) [file 11095_2019_2620_MOESM1_ESM.docx]

**Fig S1:** Polydispersity index for formulation PBD-F1

**Fig S2:** Zeta potential of PBD-F1


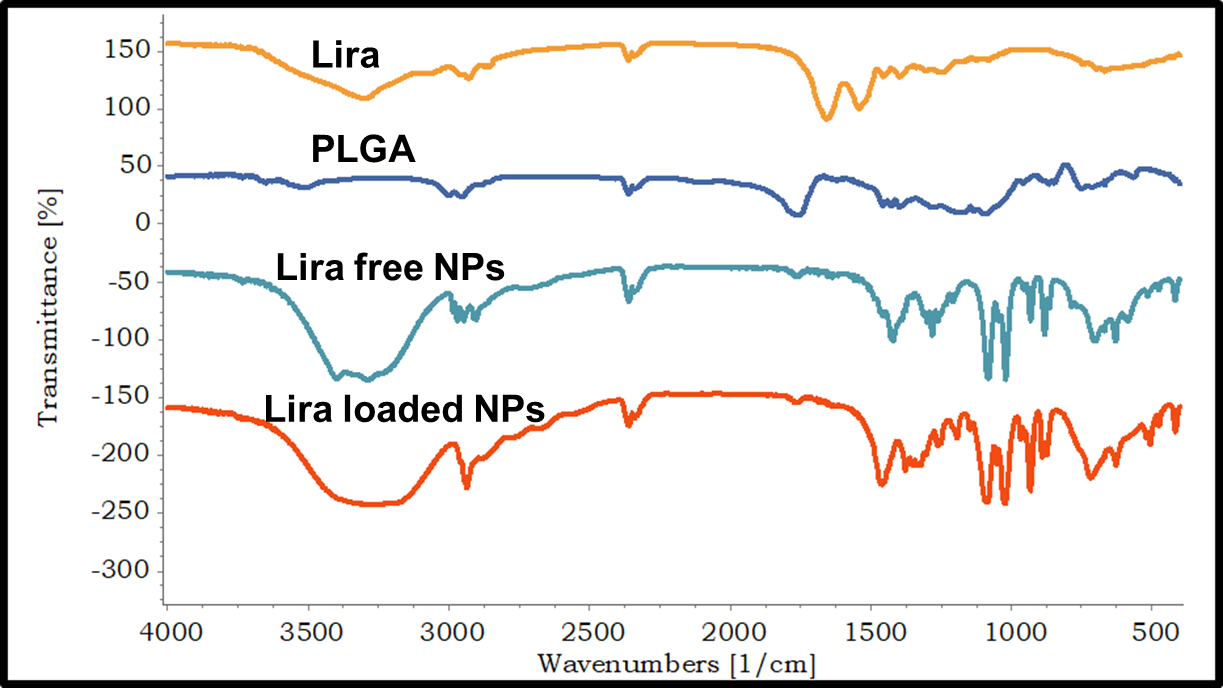


**Fig S3:** FT-IR Spectra for Lira, PLGA, Lira free NPs, Lira loaded NPs

**Lira loaded NPs**

**Lira free-NPs**

**PLGA**

**Lira**

**Fig S4:** DSC thermograms of Lira, PLGA, Lira free NPs, Lira loaded NPs


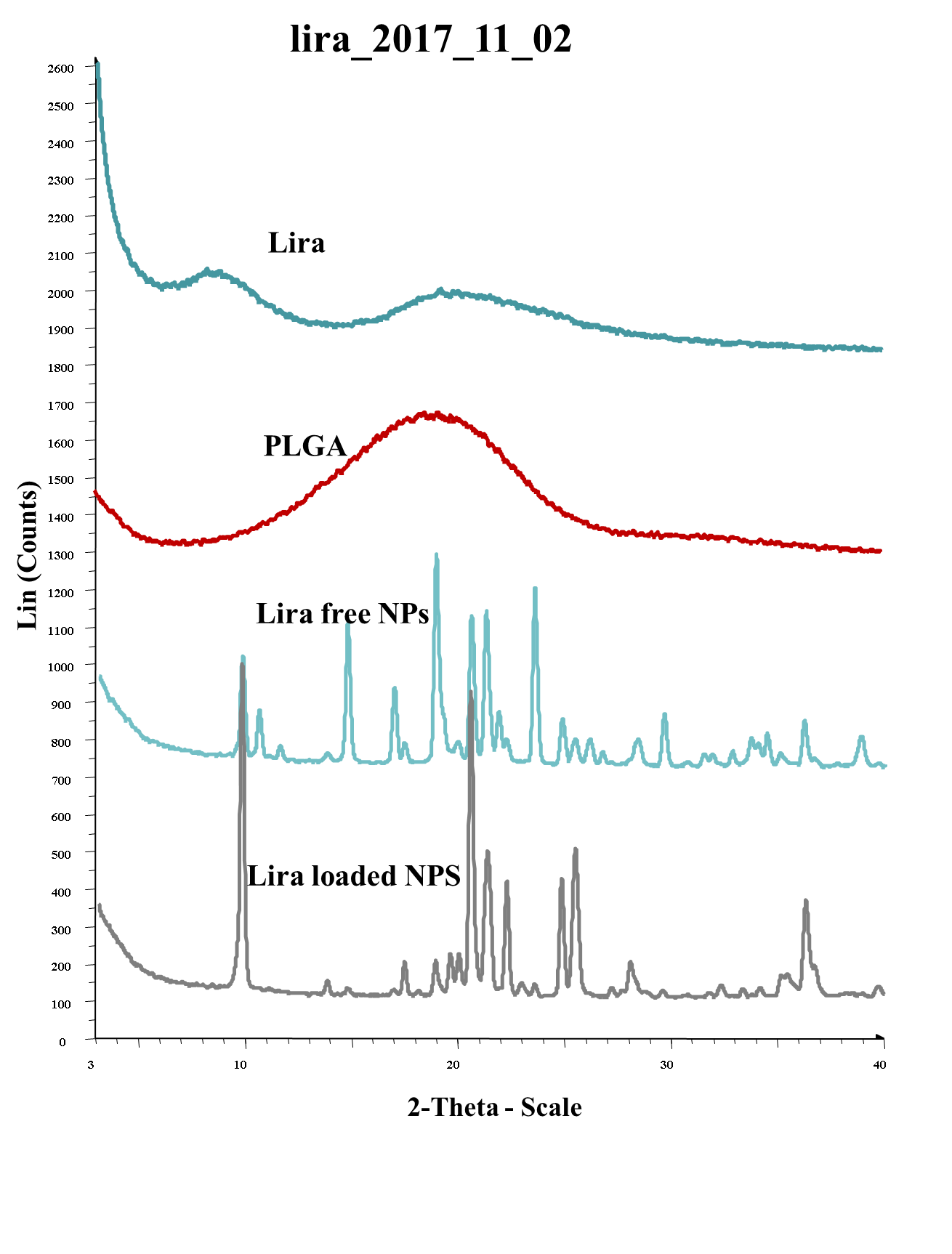


**Fig S5:** XRD diffractograms of Lira, PLGA, Lira free NPs, Lira loaded NPs
