# Supplementary material for: Synthesis and Statistical Optimization of Poly (Lactic-Co-Glycolic Acid) Nanoparticles Encapsulating GLP1 Analog Designed for Oral Delivery
Source: Pharm Res. 2019 May 13;36(7):99. doi: 10.1007/s11095-019-2620-9 (PMC6513835; doi:10.1007/s11095-019-2620-9)
Supplement: Supplementary file 2 — (DOCX 24 kb) [file 11095_2019_2620_MOESM2_ESM.docx]

**Table 1S:** ANOVA parameters for predicting mean particle size (Y1), PDI (Y2), EE(Y3) and Zeta potential (Y4)

| **Response** | **Source** | **DF** | **SS** | **MS** | **F ratio** | **P- value Prob>F*** |
| --- | --- | --- | --- | --- | --- | --- |
| **Y1** | **Model** | 8 | 12857.39 | 1605.6738 | 71.688 | <0.00001 |
|  | **Error** | 15 | 335.97 | 22.389 |  |  |
|  | **Cumulative value** | 23 | 13181.36 |  |  |  |
| **Y2** | **Model** | 8 | 0.046063 | 0.0058 | 22.669 | <0.00001 |
|  | **Error** | 15 | 0.003814 | 0.000254 |  |  |
|  | **Cumulative value** | 23 | 0.049877 |  |  |  |
| **Y3** | **Model** | 8 | 1747.522 | 218.4403 | 33.5912 | <0.00001 |
|  | **Error** | 15 | 97.544 | 6.5029 |  |  |
|  | **Cumulative value** | 23 | 1845.069 |  |  |  |
| **Y4** | **Model** | 8 | 156.7003 | 19.5875 | 16.887 | <0.00001 |
|  | **Error** | 15 | 17.3984 | 1.1599 |  |  |
|  | **Cumulative value** | 23 | 174.0987 |  |  |  |

**Table 2S:** Statistical analysis of variables effect on the mean particle size (Y1)

| **Y1: Z-AVE size** | | |
| --- | --- | --- |
|  | **Full Model** | |
| **Parameter** | **Coefficient** | **P-value** |
| **PLGA amount** | **9.5079** | ***0.0000001** |
| **Lira amount** | **1.5304** | **0.1339986** |
| **2^nd^ sonication time** | **7.7638** | ***0.0000008** |
| **PVA (%)** | **-16.9754** | ***0.0000000** |
| **Lyoprotectant type** | **-5.4721** | ***0.0000449** |
| **Lyoprotectant (%)** | **-3.5429** | ***0.0022866** |
| **W_2_/O ratio** | **-7.0971** | ***0.0000024** |

***Most significant variables**

| **Y2: PDI** | | |
| --- | --- | --- |
|  | **Full Model** | |
| **Parameter** | **Coefficient** | **P-value** |
| **PLGA amount** | **-0.0069** | **0.050614** |
| **Lira amount** | **0.0312** | ***0.000000** |
| **2^nd^ sonication time** | **0.0005** | **0.879962** |
| **PVA (%)** | **-0.0013** | **0.687862** |
| **Lyoprotectant type** | **-0.0032** | **0.346037** |
| **Lyoprotectant (%)** | **-0.0031** | **0.358505** |
| **W_2_/O ratio** | **0.0291** | ***0.000000** |

**Table 3S:** Statistical analysis of variables effect on PDI (Y2)

***Most significant variables**

**Table 4S:** Statistical analysis of variables effect on EE (Y3)

| **EE** | | |
| --- | --- | --- |
|  | **Full Model** | |
| **Parameter** | **Coefficient** | **P-value** |
| **PLGA amount** | **1.7683** | **0.003981*** |
| **Lira amount** | **1.3458** | **0.020688*** |
| **2^nd^ sonication time** | **-4.5825** | **0.000000*** |
| **PVA (%)** | **-1.4458** | **0.014082*** |
| **Lyoprotectant type** | **-2.8408** | **0.000066*** |
| **Lyoprotectant (%)** | **-5.7483** | **0.000000*** |
| **W_2_/O ratio** | **1.9150** | **0.002234*** |

***Most significant variables**

**Table 5S:** Statistical analysis of variables effect on Zeta potential (Y4)

| **Y4: Zeta Potential** | | |
| --- | --- | --- |
|  | **Full Model** | |
| **Parameter** | **Coefficient** | **P-value** |
| **PLGA amount** | **-0.0154** | **0.945019** |
| **Lira amount** | **-0.9071** | ***0.000897** |
| **2^nd^ sonication time** | **0.4496** | **0.058807** |
| **PVA (%)** | **-0.0646** | **0.772955** |
| **Lyoprotectant type** | **-0.4679** | **0.050284** |
| **Lyoprotectant (%)** | **-0.1663** | **0.461220** |
| **W_2_/O ratio** | **2.2846** | ***0.000000** |

***Most significant variables**
